# Typhoid fever in children in Africa

**DOI:** 10.1111/j.1365-3156.2008.02031.x

**Published:** 2008-04

**Authors:** Evanson Mweu, Mike English

**Affiliations:** 1 Kenya Medical Research Institute (KEMRI), Centre for Geographic Medicine ResearchNairobi, Kenya; 2 College of Health Sciences, University of NairobiNairobi, Kenya; 3 Department of Paediatrics, University of OxfordOxford, UK

**Keywords:** typhoid fever, Widal test, perception, over-treatment, Africa

## Abstract

Estimates for the year 2000 suggested that there were approximately 21.5 million infections and 200,000 deaths from typhoid fever globally each year, making the disease one of the most serious infectious disease threats to public health on a global scale. However, these estimates were based on little data, especially from Africa. Global prominence and high-profile outbreaks have created the perception in Kenya that typhoid is a common cause of febrile illness. The Widal test is used widely in diagnosis. We have reviewed recent literature, taking the perspective of a healthcare provider, to collate information on the prevalence of typhoid in children particularly, and to explore the role of clinical diagnosis and diagnosis based on a crude, but common, interpretation of the Widal test. Data suggest that typhoid in children in rural Africa is uncommon, perhaps 100 times or 250 times less common than invasive disease because of *Haemophilus influenzae* or *Streptococcus pneumoniae*, respectively. Frequent use of the Widal test may result in many hundreds of over-treatment episodes for every true case treated and may perpetuate the perception that typhoid is common. Countries such as Kenya need better bacterial disease surveillance systems allied to better information for healthcare providers to promote appropriate decision-making on prevention and treatment strategies.

## Introduction

Typhoid fever, also known as enteric fever, is caused by the Gram-negative bacterium *Salmonella enterica* serovar Typhi. The disease is mainly associated with low socio-economic status and poor hygiene, with human beings the only known natural hosts and reservoir of infection. Estimates for the year 2000 suggest that there are approximately 21.5 million infections and 200,000 deaths from typhoid fever globally each year (Crump *et al.* 2004; Bhan *et al.* 2005; Bhutta 2006). It is thus considered one of the most serious infectious disease threats to public health on a global scale, with particular concern over the rapid and widespread emergence of resistance to multiple antibiotics (Akinyemi *et al.* 2005; Feng 2000). The global concern over typhoid is reflected in perceptions that typhoid is a common and serious disease among children and adults in Kenya, where highly publicised outbreaks have strengthened this view among the public and health professionals. One consequence is the common use of the Widal test to ‘screen’ febrile children and adults in inpatient and outpatient settings, as few centres have the capacity to perform blood or bone marrow culture, the accepted gold standard diagnostic tests (Chart *et al.* 2000; Willke *et al.* 2002).

The global burden of disease estimates for typhoid were based on a total of 22 community-based incidence studies with 19 from continents other than Africa and only three from Africa. On the basis of these data and a prediction rule based on climatic and socio-economic features, continental estimates of disease burden were derived (Crump *et al.* 2004). These estimates suggested a moderate incidence of typhoid of 10–100 cases/100,000 person years in most African countries, with the incidence highest in childhood. In East Africa, incidence was estimated at 39/100,000 person years. Recently, increasing amounts of data on the prevalence of different pathogens found in sick children presenting to health facilities in Africa have been reported. These data have considerably raised the profile of non-typhoidal salmonella infections (Graham *et al.* 2000). To date, however, these data have not been used to examine the burden of disease attributable to typhoid. We hypothesised that insights into the relative burden of disease attributable to typhoid, although not the exact burden, could be gained by examining recent facility-based information on the aetiology of invasive bacterial infections. If typhoid is a major, widespread pathogen, it should be frequently observed in situations where other major childhood pathogens, for example, *Streptococcus pneumonia* and *Haemophilus influenzae* are frequently observed. From the perspective of the health system and healthcare provider, we were also interested to review pertinent data on the usefulness of clinical case definitions and the Widal test in the diagnosis of typhoid in children in an African setting.

## Methods

### Searches

Potential studies for inclusion were identified through direct searches of MEDLINE database through PubMed. Searches were structured around our three principal questions on typhoid: (1) Epidemiology, with a particular emphasis on prevalence in routine clinical settings. When articles were identified, they were classified as those that provided information on prevalence of typhoid fever in (a) *Unselected* populations of sick children or adults presenting to health facilities with minimal selection criteria for inclusion into the study; for example, an investigation of all febrile outpatient or inpatient cases; (b) *selected* groups/populations of children or adults with specific criteria for inclusion into the study; for example, investigation of only those with prolonged fever and a clinical diagnosis of possible typhoid fever; (c) *case-series*. In this category, we included those studies that reported only culture-positive cases of typhoid as a proportion of all cases of confirmed invasive bacterial disease. Studies included were further categorised as representing Asian or African populations. (2) Clinical diagnosis or clinical presentation of typhoid fever in children. (3) Performance evaluation of the Widal test.

Broad MeSH terms were used for searches (Table 1). The search on studies contributing to an understanding of epidemiology was restricted to publications from 1996 to date using both the broad sensitive and narrow specific filters that are included in PubMed's clinical queries function (Haynes *et al.* 1994). Newly-identified publications were added to those listed and reviewed by Crump *et al.* (2004) in their global burden of disease estimates. All titles and abstracts were reviewed and selections made on the basis of relevance to the precise study questions. Supplementary searches of the bibliographies of retrieved articles and by contacting experts in the topic area were performed. The authors of recent published and unpublished data from Africa describing the pattern of bacteraemia in children were contacted for specific information on the prevalence of typhoid fever within the population studied (Berkley *et al.* 2005; Brent *et al.* 2006; Enwere *et al.* 2006; Roca *et al.* 2006).

**Table 1:** Summary of the main search terms and results

Search	Search terms	No. of hits/ abstracts reviewed	No. of papers included
1	‘Typhoid fever’ (MeSH) OR ‘paratyphoid fever’ (MeSH) OR ‘salmonella infections’ (MeSH) limits: published in the last 10 years, clinical trial, meta-analysis, practice guideline, randomised controlled trial, review, humans	538	4
2	[(Typhoid fever OR salmonellosis OR paratyphoid fever OR enteric fever) AND epidemiology] AND	162	2
3	‘Typhoid fever’ (MeSH) AND ‘Diagnosis’ (MeSH) AND ‘Child’ (MeSH)	377	5 extra (not in 1 and 2)
4	‘Signs and symptoms’ (MeSH) AND ‘typhoid fever’ (MeSH) AND ‘child’ (MeSH)	142	1 extra (not in 1,2 and 3)
5	(Bacteraemia AND child) AND (run under diagnosis and the narrow specific filter)	64	1 extra
6	(Bacteraemia AND child) AND (run under aetiology and the narrow specific filter)	169	1 extra
Total		1290	14

### Exploring the consequences of widespread use of the Widal test

To explore the possible consequences of widespread use of the Widal test, we used test performance characteristics (sensitivity and specificity) reported in the literature based on a single test titre of ≥1:100 for either the O or H antigen. In simple models, these test performance parameters were applied to a theoretical population of 10,000 febrile African children presenting for clinical care whose true prevalence of typhoid varied between 0.1% and 5% based on our literature review. We thus calculated the numbers of true positives and false positives in a range of scenarios by varying prevalence, sensitivity and specificity. By dividing the number of false positives by the number of true positives we computed, a ratio that we shall call the *over-treatment ratio* (OTR). This number is a measure of the number of children treated without proven disease for every one child treated who has the disease as a result of relying on the Widal test for diagnosis.

## Results

### Prevalence of typhoid

Fifteen articles providing information on prevalence in Africa were included in the review (Table 2). Ten reported blood culture data from populations fulfilling minimal or no selection criteria (Table 2), three reported data on selected patient groups while two took as their denominator only positive blood cultures. We considered the 10 studies on unselected populations as providing the most appropriate data on the likely prevalence of typhoid among sick children presenting with at least a moderately severe febrile illness. Of these, only one reports data exclusively on outpatients and one data from a mixed outpatient and inpatient population(Brent *et al.* 2006; Enwere *et al.* 2006). In the 10 studies, the reported prevalence ranges from 0% to 4.23%, with only the study carried out in Egypt (Crump *et al.* 2003) reporting prevalences of more than 1% (4.23%) (Lepage *et al.* 1987; Walsh *et al.* 2000; Bahwere *et al.* 2001; Gordon *et al.* 2001; Wilson *et al.* 2003; Berkley *et al.* 2005; Brent *et al.* 2006; Enwere *et al.* 2006; Roca *et al.* 2006). In studies on *selected populations*, that were in general much smaller, one reported a prevalence of 0% (20), one a prevalence of 9% (Cheesbrough *et al.* 1997) and the last a prevalence of 22% (Ammah *et al.* 1999). While the data reported from case-series confirm the presence of typhoid fever in these settings, they provide no clear information on the burden of disease.

**Table 2:** Estimated prevalence of typhoid fever in African, facility-based studies

Citation	Country/setting	Study design	Inclusion criteria	Sample size (n)	Positive culture	Results (*S. typhi*)	*S. typhi*% of +ve cultures	% of *S. typhi* In screened patients
Brent *et al.* (2006) and A. Scott, unpublished data	Kenya (rural population)	Prospective study	Under 5 years; outpatients, febrile or with IMCI type disease, excluded were children admitted to the hospital within the previous 10 days	1093 children	22	0/22	0.00	0.00 (0/1093)
Enwere *et al.* (2006) and R. Adegbola, unpublished data	Gambia (rural population)	Prospective study	2–29 months. In and outpatients, with signs of infection and a temperature of ≥38°C. Carried out as part of a pneumococcal vaccine trial	7,369 specimens	355 (blood, CSF, lung aspirate)	0/355	0.00	0.00
Roca *et al.* (2006) and A. Roca, unpublished data	Mozambique (rural population)	Prospective study	<5-year-old, inpatients, febrile. Cultures done for all <2-year-olds admitted and for older children with fever of >39°C or <15-year-olds with neurological signs	10,702 children	810 (blood, CSF)	1/810	0.12	0.009
Berkley *et al.* (2005) and A. Scott, unpublished data	Kenya (rural population)	Prospective study	Under 13 years; all admitted except those for elective procedures or accidents	19,339 children	1094 (blood)	1/1094	0.09	0.0052 (1/19339)
Crump *et al.* (2003)	Egypt (mixed population)	Prospective study	≥6 months old, fever for ≥3 days	449 persons	36 (blood)	19/36	52.78	4.23 (19/449)
Wilson *et al.* (2003)	Malawi (mixed population)	Prospective study	Both children and adults, admitted	31,035 cultures	4529 (blood, CSF)	34/4529 (*all from adults*)	0.75	0.001 (34/31,035)
Bahwere *et al.* (2001)	Zaire (rural population)	Prospective study	All children; On admission *(whether febrile or not*)	932 children	124 (blood)	2/124	1.6	0.2 (2/932)
Gordon *et al.* (2001)	Malawi (mixed population)	Prospective study	All febrile, adult admissions	2789 persons	449 (blood)	12/449	2.67	0.43 (12/2789)
Walsh *et al.* (2000)	Malawi (mixed population)	Prospective study	All febrile children, without an obvious cause for fever, 87% aged <5 years	2123 cultures	365 (blood)	15/365	4.1	0.7 (15/2123)
Lepage *et al.* (1987)	Rwanda (mixed population)	Prospective study	Under 15-year-olds; febrile ≥39°C, outpatients and inpatients within 24 h of admission, excluded were children admitted to the hospital within the preceding 3 months and those with measles up to 10 days after onset of rash	14032 children	112 (blood)	47/112	42	0.33 (47/14032)
Nsutebu *et al.* (2003)	Cameroon (urban population)	Prospective study	Febrile patients with typhoid fever as provisional diagnosis, ≤4-year-olds and those with a definite diagnosis other than typhoid were excluded	200 patients	4 (blood, stool)	0/4	0.00	0.00 (0/200)
Ammah *et al.* (1999)	Cameroon (urban population)	Prospective study	All febrile cases combined with any other symptom suggestive of typhoid fever, age 4–75 years	200 persons (53 aged ≤5 years)	133 (blood, stool)	44/133	33.08	22.00 (44/200)
Cheesbrough *et al.* (1997))	Zaire (rural population)	Prospective study	1–16-year-olds, that fitted into a preset clinical case definition of salmonella bacteraemia, in and outpatients	120 children	55 (blood, stool)	11/55	20.00	9.17 (11/120)
Issack *et al.* (2005)	Mauritius	Retrospective study	Laboratory confirmed typhoid fever cases	25 hospital records		25 cases of typhoid fever in 8 years	–	
Green *et al.* (1993)	Zaire	Prospective study	≤5 years, febrile, outpatient, clinically suspected typhoid fever	–	206 (blood, CSF, joint aspirate)	34/206	16.5	–

Twelve studies from Asia and the Indian sub-continent were identified for comparison as this region is also reported to have a high prevalence of typhoid fever (Table 3). Six reported blood culture data from populations in whom minimal or no selection criteria were applied (included in this group are studies with no selection criteria for children aged <5 years but minimal criteria for those aged ≥5 years), four studies reported data from selected populations and two were case-series. Among studies which considered the unselected group, only 2/6 report prevalences >1%, with 5.51% prevalence in Dhaka, Bangladesh and 6.1% in Malaysia (Choo *et al.* 1993; Sinha *et al.* 1999; Feng 2000; Brooks *et al.* 2005; Siddiqui *et al.* 2006). In studies on selected populations, the reported prevalence was between 8.6% and 71.5% (Wain *et al.* 1998; Vollaard *et al.* 2004). The high figures in this group may be attributed to highly restricted inclusion criteria. For example, the prevalence of 71.5% was among patients with clinically suspected, uncomplicated typhoid fever who had fever for ≥6 days with no obvious focus of infection, a negative malaria blood smear, abdominal discomfort and change of bowel habit.

**Table 3:** Estimated prevalence of Typhoid Fever in studies from Asia and the Indian sub-continent

Citation	Country/setting	Study design	Inclusion criteria	Sample size	Positive cultures	Results (*S. typhi*)	*S. typhi*% of +ve cultures	%*S. typhi* in screened patients
Siddiqui *et al.* (2006)	Pakistan (urban)	Prospective study	Under 16 years, febrile for ≥72 h, no localising signs	11,668 children	123 (blood)	42/123	34.15	0.36 (42/11668)
Sur *et al.* (2006)	India (urban)	Prospective study	Fever for ≥3 days, irrespective of age, outpatients	60,452 persons	95 (blood)		–	0.16 (95/60452)
Brooks *et al.* (2005)	Bangladesh (urban)	Prospective study	<5 years with fever ≥38°C, >5 years with fever for ≥3 days, outpatients	889 persons	65 (blood)	49/65	75.38	5.51 (49/889)
Sinha *et al.* (1999))	India (urban)	Prospective study	All febrile cases, ≤5 years, fever ≥3 days for those aged 5–39 years. Residents of study area, outpatients	8172	1217 (blood)	63/1217	5.18	0.78 (63/8172)
Choo *et al.* (1993)	Malaysia (mixed)	Prospective study	1 month, 12 years, febrile, admitted	2382 children	145 (blood, stool, urine)	–	–	6.1 (145/2382)
Feng (2000))	Vietnam (rural)	Prospective study	Adults and children, febrile for ≥3 days, fever of ≥38.5°C, presenting to clinics and hospitals	28,329 persons	56			0.2 (56/28329)
Nizami *et al.* (2006a)	Pakistan (mixed)	ProspectiveStudy	2–14 years; febrile, clinically suspected typhoid fever	214 children	26 *S. typhi* (blood) 16 (PCR)	–	–	18.69 (40/214)
Vollaard *et al.* (2004)	Indonesia	Case-control study	Febrile persons, ≥3 days fever; all ages, in and outpatients	1019 persons *(no age stratification in report*)	125 (blood)	88/125 *S. typhi*	70.40	8.64 (88/1019)
Bhutta and Mansurali (1999)	Pakistan (urban)	ProspectiveStudy	Children, febrile, ambulatory, clinically suspected. Typhoid fever	97 children	46 (blood, Bone marrow in those who had used antibiotics for ≥72 h)	–	–	47.42 (46/97)
Wain *et al.* (1998)	Vietnam (urban)	Prospective study	Adults and children, admitted with suspected enteric fever; excluded were patients with complicated typhoid or those effectively treated for typhoid.	515 persons	375 (blood)	369/375	98.40	71.65 (369/515)
Walia *et al.* (2005a)	India	Retrospective study	Culture-confirmed enteric fever; in and outpatients; all ages	–	377	304/377	80.64	–
Wong *et al.*; (1994)	China	Retrospective study	Records of salmonella isolations by the clinical laboratory in all ages	–	5328 (stool, blood, urine, surgical specimen)	351/5328	6.59	–

### Clinical presentation

The common signs and symptoms present in patients (both adults and children) with culture-proven typhoid fever from nine published studies are summarised in Table 4. The signs that are most commonly reported are fever followed by diarrhoea, vomiting, abdominal pain, headache, splenomegaly, anorexia and hepatomegaly. There is no obvious difference in clinical presentation of typhoid between Asia and Africa. We are only aware of two studies that have explored the performance of a clinical case definition for typhoid fever, one study in Turkey (Hosoglu *et al.* 2006) and one in Indonesia (Vollaard *et al.* 2005). In Turkey, seven variables were considered to be highly indicative of typhoid infection: Age <30 years, abdominal distension, confusion, relative bradycardia, typhoid tongue, a positive Widal test and leucopoenia. In *post-hoc* analyses, the authors created a scale based on these features and reported an optimum diagnostic performance of sensitivity 86.2%, specificity 76.9%, positive predictive value (PPV) 78.7%, and negative predictive value (NPV) 84.0% in their study population aged ≥15 years who presented at hospital with a febrile illness. The Indonesian study, however, concluded that no symptom and sign combination performed adequately to be useful in clinical practice for identifying typhoid (Vollaard *et al.* 2005).

**Table 4:** Prominent signs and symptoms in culture confirmed cases of typhoid fever

	Nsutebu *et al.* (2003)	Ammah *et al.* (1999)	Walia *et al.* (2005)	Mathura *et al.* (2005)	Kumar *et al.* (2002)	Papaevangelou *et al.* (2004)	Ispahani *et al.* (2000)	Sinha *et al.* (1999)	Siddiqui *et al.* (2006)
Fever	+	+	+	+	+	+	+	+	+
Anorexia	+	+	−	−	−	−	−	−	+
Vomiting	+	+	+	+	−	+	+	+	+
Hepatomegaly	−	−	+	−	+	−	+	−	−
Diarrhoea	+	+	+	+	+	+	+	+	+
Abdominal pain	+	+	+	+	−	−	+	−	+
Splenomegaly	−	−	+	+	+	−	+	+	−
Constipation	−	+	−	+	+	−	−	−	+
Headache	+	+	−	+	−	−	+	−	+
Intestinal perforation	−	−	−	−	+	−	−	−	−
Myalgia	−	+	−	−	−	−	−	−	−

### Performance of the Widal test

We identified four studies that report the sensitivity and specificity of a single pre-treatment Widal test for detecting typhoid, compared with blood and/or bone marrow culture as the gold standard. Data on the performance of newer rapid diagnostic tests (Tyhidot®, Typhidot-M®, Tubex®) are not reported as these are not routinely available in our setting. Studies frequently report sensitivities and specificities for a variety of threshold titres with, predictably, a general decline in sensitivity and increase in specificity as the titre required for diagnosis is increased (Table 5). The control populations used for comparison purposes in the reported studies were generally sick patients who were diagnosed as not having typhoid fever either clinically or by culture. While the conclusion of many authors is that locally appropriate threshold titres need to be established in each setting before the Widal test can be reliably used, it is our experience that a threshold titre of ≥100 for either or both the O or H antigens is commonly regarded as ‘significant’ and indicative of clinical typhoid in many Kenyan settings. The sensitivity and specificity of a single pre-teatment Widal test at this titre (≥100) are reported to vary between 50% and 90% (Table 5).

**Table 5:** Reported sensitivity and specificity of the Widal test using the tube agglutination technique

Antigen combination	Citation	Population studied	Titre (≥)	Sensitivity %	Specificity %
O and H	Bhutta and Mansurali (1999)	Children (no age cut-off) (46 cases)(26 controls)	100	55	81
O or H	Olsen *et al.* (2004)	Patients ≥3 years (59 cases) (20 controls)	100	64	76
O or H	Parry *et al.* (1999)	Children <15 years, adults ≥15 years(1400 cases) (555 controls)	100	92	83
			200	66	97
			400	36	99
O or H	Choo *et al.* (1993)	Children, 1 month,12 years, admitted.(145 cases) (2064 controls)	80	72.6	92
			160	72.4	93.9
			320	49.7	95.9

**Figure 1:**
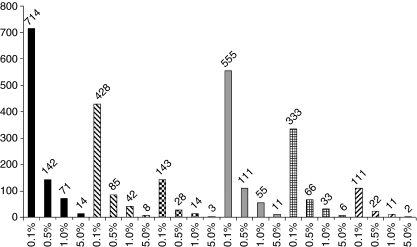
Calculated over-treatment ratios (*y*-axis) at different levels of typhoid prevalence (x-axis) for a presenting population of 10,000 sick people. (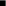) Series with sensitivity 50% and specificity 70%. (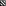) Series with sensitivity 70% and specificity 70%. (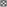) Series with sensitivity 90% and specificity 70%. (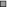) Series with sensitivity 50% and specificity 90%. (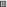) Series with sensitivity 70% and specificity 90%. (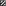) Series with sensitivity 90% and specificity 90%.

Based on the published data, our models indicate that the over-treatment ratio (OTR) in the worst case scenario would be 999 when the Widal test has a sensitivity of 50% and a specificity of 50% if the true prevalence of typhoid in the presenting population is 0.1%. In the best case scenario, the OTR is 2.1, when both sensitivity and specificity are 90% and a true typhoid prevalence of 5% is assumed (Figure 1, where specificity is fixed at 70% or 90%).

## Discussion

Typhoid fever has a high profile as a life-threatening disease for which treatment is becoming increasingly difficult because of the emergence of strains resistant to multiple antibiotics (Feng 2000; Akinyemi *et al.* 2005; Bhan *et al.* 2005; Chowta & Chowta 2005). In Kenya, widely publicised disease outbreaks have led to calls for mass vaccination and it is our experience that clinicians consider the disease to be reasonably common among febrile adults and children presenting to them for care. Global estimates of the burden of disease for typhoid reinforce this view. However, the current global estimates are based on very limited data, especially from Africa. The data we present show considerable variability in the prevalence of confirmed typhoid between settings and perhaps suggest a significant urban/rural disparity worthy of further investigation.

While there remain very few studies of the incidence of typhoid, we reasoned that insight into the burden of disease could also be gained from the increasing number of studies reporting the prevalence of serious bacterial infections in minimally selected patient populations, particularly children, presenting to clinical settings. In many of these studies, a comparison with the reported prevalence of alternative pathogens causing severe disease such as *Haemophilus influenzae* or *Streptococcus pneumoniae* can be made (Berkley *et al.* 2005; Enwere *et al.* 2006; Roca *et al.* 2006). Where these reports provide estimates of incidence, albeit an underestimate related to the passive nature of disease identification, the relative incidence of typhoid can be estimated. Thus, in one well-characterised Kenyan population, if the true incidence of typhoid in children was 39/100,000 cyo, the figure assumed for East Africa (Crump *et al.* 2004), then we might expect two isolates of *S. typhi* for every three of *H. influenzae* and one isolate of *S. typhi* for every three of *S. pneumoniae* (Berkley *et al.* 2005). In fact, only one isolate was identified compared with 136 for *H. influenzae* and 282 for *S. pneumoniae*, a finding similar to that observed in The Gambia (Enwere *et al.* 2006; and R. Adegbola, unpublished data) and Mozambique (Roca *et al.* 2006, and A. Roca, unpublished data). On the basis of the Kenyan data, the incidence of culture-proven typhoid in rural children may therefore be lower than 1/100,000 cyo although this almost certainly is an underestimate, given the use of passive case detection and the insensitivity of blood culture.

There have been few attempts to establish simple clinical case definitions for typhoid fever to help in case identification at presentation to health facilities. In one of the two studies we are aware of, the results were so poor that the authors did not consider clinical diagnosis adequate(Vollaard *et al.* 2005). The other study produced a complex scoring system requiring two laboratory-based parameters (including the Widal test) and was undertaken in adults in a country without common alternative diagnoses prevalent in African children such as malaria and HIV infection (Hosoglu *et al.* 2006). Given the non-specific nature of the clinical presentation, it would appear that further work in this area is unlikely to produce an effective, simple clinical diagnostic approach pointing to a clear need for an accurate, simple and inexpensive diagnostic test.

Unfortunately, the most widely used diagnostic test, the Widal test, performs very poorly as a screening test for febrile children especially in areas of low prevalence likely to be typical of much of rural Africa. Thus, routine use of the Widal test in clinical practice in low prevalence settings will result in an unacceptably high number of children treated inappropriately, resulting in widespread use of antibiotics usually preserved as second- or third-line therapies with the attendant risk of exacerbating problems with antimicrobial resistance. Even in Asia where there is a suggestion that incidence rates and prevalence of disease in clinical settings may be slightly higher, the consequences of unnecessary treatment may be pronounced.

## Implications

The findings in this review show that there is need for better data on typhoid fever in children in Africa. This data would be best generated by effective, broadly based public health surveillance systems that could capture data on a range of serious bacterial diseases in urban and rural settings. Few or no such systems exist in the poorer countries of Africa where disease burdens are the highest. This paucity of information contributes to ill-informed discussions on priority interventions, particularly vaccines. It is also apparent that the Widal test is not only unlikely to be helpful in many African settings, it is most commonly likely to be misleading. Missed diagnoses and worsening antibiotic resistance may result, although the higher prevalence of non-typhoidal salmonellae (NTS) makes it likely that on some occasions a child falsely diagnosed as having typhoid will, by chance, receive an appropriate treatment for NTS (Graham *et al.* 2000). While newer, improved diagnostics are therefore urgently required, it is clear that they will have to be highly sensitive and specific (>90%) to truly contribute to better health in settings where access to highly trained health workers with adequate time is rare and disease prevalence is low.
